# Supplementary material for: Shared computational principles for language processing in humans and deep language models
Source: Nat Neurosci. 2022 Mar 7;25(3):369–80. doi: 10.1038/s41593-022-01026-4 (PMC8904253; doi:10.1038/s41593-022-01026-4)
Supplement: Supplementary file 1 — Appendices I & II [file 41593_2022_1026_MOESM1_ESM.pdf]

nature portfolio

https://doi.org/10.1038/s41593-022-01026-4

Supplementary information

# Shared computational principles for language processing in humans and deep language models

In the format provided by the authors and unedited

Appendix I - Decoding Model Details

*The size of the layer is dependent on the number of electrons included in the fold (5 folds over all). The number of electrodes ranged from 114-132, and the total number of parameters ranged from 219,670-228,790.

Layer (type)	Output Shape	Param #
input_1 (InputLayer)	[(None, 10, *114-132X)]	0
conv1d (Conv1D)	(None, 8, *114-132)	55680-64 800
activation (Activation)	(None, 8, *114-132)	0
batch_normalization (BatchNorn)	(None, 8, *114-132)	640
dropout (Dropout)	(None, 8, *114-132)	0
max_pooling1d (MaxPooling1D)	(None, 4, *114-132)	0
conv1d_1 (Conv1D)	(None, 3, *114-132)	51200
activation_1 (Activation)	(None, 3, *114-132)	0
batch_normalization_1 (Batch	(None, 3, *114-132)	640
dropout_1 (Dropout)	(None, 3, *114-132)	0
locally_connected1d (Locally	(None, 2, *114-132)	102720
batch_normalization_2 (Batch	(None, 2, *114-132)	640
activation_2 (Activation)	(None, 2, *114-132)	0
global_max_pooling1d (Global	(None, *114-132)	0

dense (Dense)	(None, 50)	8050
layer_normalization (LayerNo	(None, 50)	100
**Total parameters**		***219,670-228,790**
**Trainable parameters**		***218,710-227,830**
**Non-trainable parameters**		**960**

- Learning rate: 0.00025
- Batch size: 256
- Convolutional layers L2 regularization alpha: 0.003
- Dense layer L2 regularization alpha: 0.0005
- Dropout probability is 21%
- Weights averaged over last 20 epochs before early stopping
- Trained for a maximum of 1500 epochs with patience of 150 epochs

We used a hyperparameter search to choose depth, batch size, learning rate, patience, and convolutional filter. [15]

Appendix II - Word List

a	called	have	make	very	think	who
about	camera	he	me	public	this	wikipedia
after	case	him	monkey	really	thought	with
all	copyright	his	my	right	to	would
an	could	how	next	said	twenty	yeah
and	court	human	no	saw	two	year
andrew	david	i	not	say	uh	you
animal	day	if	now	see	um	your
are	did	in	of	should	up	
argument	do	into	on	so	very	
around	domain	is	one	sued	wa	
at	even	it	or	take	wales	
attorney	first	judge	other	that	way	
be	for	just	out	the	we	

because	friend	know	over	their	well
been	from	law	own	them	were
before	get	lawyer	people	then	what
being	got	legal	photo	there	when
but	ha	like	photograph	these	where
by	had	look	picture	they	which
