# Supplementary material for: Shared computational principles for language processing in humans and deep language models
Source: Nat Neurosci. 2022 Mar 7;25(3):369–80. doi: 10.1038/s41593-022-01026-4 (PMC8904253; doi:10.1038/s41593-022-01026-4)
Supplement: Supplementary file 2 — Reporting Summary [file 41593_2022_1026_MOESM2_ESM.pdf]

nature research

Corresponding author(s): Ariel Goldstein

Last updated by author(s): 2021/12/19

Reporting Summary

Nature Research wishes to improve the reproducibility of the work that we publish. This form provides structure for consistency and transparency in reporting. For further information on Nature Research policies, see our Editorial Policies and the Editorial Policy Checklist.

Statistics

For all statistical analyses, confirm that the following items are present in the figure legend, table legend, main text, or Methods section.

n/a	Confirmed	
☐	☒	The exact sample size (*n*) for each experimental group/condition, given as a discrete number and unit of measurement
☐	☒	A statement on whether measurements were taken from distinct samples or whether the same sample was measured repeatedly
☐	☒	The statistical test(s) used AND whether they are one- or two-sided<br>*Only common tests should be described solely by name; describe more complex techniques in the Methods section.*
☒	☐	A description of all covariates tested
☐	☒	A description of any assumptions or corrections, such as tests of normality and adjustment for multiple comparisons
☐	☒	A full description of the statistical parameters including central tendency (e.g. means) or other basic estimates (e.g. regression coefficient) AND variation (e.g. standard deviation) or associated estimates of uncertainty (e.g. confidence intervals)
☐	☒	For null hypothesis testing, the test statistic (e.g. F, t, r) with confidence intervals, effect sizes, degrees of freedom and P value noted<br>*Give P values as exact values whenever suitable.*
☒	☐	For Bayesian analysis, information on the choice of priors and Markov chain Monte Carlo settings
☒	☐	For hierarchical and complex designs, identification of the appropriate level for tests and full reporting of outcomes
☒	☐	Estimates of effect sizes (e.g. Cohen's *d*, Pearson's *r*), indicating how they were calculated

Our web collection on statistics for biologists contains articles on many of the points above.

Software and code

Policy information about availability of computer code

Data collection	Behavioral data were collected Mechanical Turk.
Data analysis	Data were preprocessed using Matlab 2019b and The Fieldtrip toolbox. Data was analyzed using python packages are specified here https://github.com/hassonlab/247-main/blob/main/env.yml. Brain plots were done using toolbox for MATLAB available at (https://github.com/HughWXY/ntools_elec). All scripts for analyses are available at:All the scripts for analyses can be found at: https://github.com/orgs/hassonlab/repositories

For manuscripts utilizing custom algorithms or software that are central to the research but not yet described in published literature, software must be made available to editors and reviewers. We strongly encourage code deposition in a community repository (e.g. GitHub). See the Nature Research guidelines for submitting code & software for further information.

Data

Policy information about availability of data

All manuscripts must include a data availability statement. This statement should provide the following information, where applicable:

- Accession codes, unique identifiers, or web links for publicly available datasets
- A list of figures that have associated raw data
- A description of any restrictions on data availability

We will make the data available 6 month after paper publication. Pending anonymization process.

Field-specific reporting

Please select the one below that is the best fit for your research. If you are not sure, read the appropriate sections before making your selection.

☒ Life sciences ☐ Behavioural & social sciences ☐ Ecological, evolutionary & environmental sciences

For a reference copy of the document with all sections, see nature.com/documents/nr-reporting-summary-flat.pdf

Life sciences study design

All studies must disclose on these points even when the disclosure is negative.

Sample size	No statistical methods were used to pre-determine sample sizes but our final sample sizes are similar to those reported in previous publications. For exmaple, Michelmann et. al. (2021). Moment-by-moment tracking of naturalistic learning and its underlying hippocampo-cortical interactions. Nature communications.
Data exclusions	One patient was removed according pre-established exclusion criteria of excessive epileptic activity .
Replication	Pre-existing behavioral data that were analyzed in this study were replicated in a new sample. The neural results are replicated at the single patient level (which can be considered as replication). No failed replications have be found.
Randomization	Randomization was not applicable as patients freely hear a strory when their neural activity is measured. Behavior pilot for predictability scores also did not require randomization
Blinding	Blinding was not relevant in this study, because participants and experimenter do not directly interact in online-experiments. Patients were not assigned to conditions.

Reporting for specific materials, systems and methods

We require information from authors about some types of materials, experimental systems and methods used in many studies. Here, indicate whether each material, system or method listed is relevant to your study. If you are not sure if a list item applies to your research, read the appropriate section before selecting a response.

Materials & experimental systems

n/a	Involved in the study	
☒	☐	Antibodies
☒	☐	Eukaryotic cell lines
☒	☐	Palaeontology and archaeology
☒	☐	Animals and other organisms
☐	☒	Human research participants
☐	☒	Clinical data
☒	☐	Dual use research of concern

Methods

n/a	Involved in the study	
☒	☐	ChIP-seq
☒	☐	Flow cytometry
☒	☐	MRI-based neuroimaging

Human research participants

Policy information about studies involving human research participants

Population characteristics	Population characteristics Nine epilleptic patients who volunteered to participate in the study (5 female; 20–48 years old); For the behavioral experiment we used Mechanical Turk, no details about age and gedner were collected.
Recruitment	Behavioral participants were recruited via Amazon Mechanical Turk. They were payed 10$ for their participation. Thier age and gender were not collected. The quality of behavior is assessed an no self selection criteria was found relevant. Patients were recruited via the Comprehensive<br>Epilepsy Center of the New York University School of Medicine. Patients are asked whehte they want to listen to a podcast during their time in the hospital and are consenting that the neural data will be used for analyses. No relevent self selection is known with this regard.
Ethics oversight	Princeton University and New York University School of Medicine respective Institutional Review Boards approved the studies. Board/committee (Princeton university IRB) that approved the protocol: Daniel Notterman (Chair), Calvin Chin,Elizabeth Davis,Patricia Fernandez-Kelly,Michael Graziano, Kyle Jamieson, Jacqueline Kariithi, Vikki Lovvoll, Brandt McCabe, Chad Pettengill, Pascale Poussart, Naila Rahman, Rusty Reeves, Carrie Siegler, Jordan Taylor, Rory Truex

Note that full information on the approval of the study protocol must also be provided in the manuscript.

Clinical data

Policy information about clinical studies

All manuscripts should comply with the ICMJE guidelines for publication of clinical research and a completed CONSORT checklist must be included with all submissions.

Clinical trial registration	n.a.
Study protocol	n.a.
Data collection	n.a.
Outcomes	n.a.
